# Terahertz Photonic Crystal Fiber Sensor for Cancer Cell Detection: Performance Analysis and Discrimination

**DOI:** 10.1002/cam4.71024

**Published:** 2025-07-07

**Authors:** Kayab Khandakar, Md. Omar Faruk, A. H. M. Iftekharul Ferdous, Md. Naimur Rahman Naim

**Affiliations:** ^1^ Electrical and Electronic Engineering Department Pabna University of Science and Technology Pabna Bangladesh

**Keywords:** confinement loss (CL), effective area (EA), effective material loss (EML), numerical aperture (NA), refractive index (RI), relative sensitivity (RS), spot size

## Abstract

**Background:**

Cancer cells exhibit significant heterogeneity, making their detection a challenging task.

**Methods:**

In this study, a unique Photonic Crystal Fiber (PCF) sensor with a circular core design and curved trapezoidal cladding is presented. It is especially designed to detect cancer cells efficiently.

**Results:**

The proposed sensor demonstrates exceptional performance, achieving a maximum relative sensitivity (RS) of 99.71% for breast cancer type II cells, along with similarly high sensitivities for cervical, adrenal gland, skin, blood, and breast cancer type I cells. Key performance parameters highlight its accuracy in detecting cancer cells via minute changes in refractive index (RI), including ultra‐low confinement loss (CL) of up to 6.69 × 10^−13^ dB/cm and effective material loss (EML) as low as 0.00161 cm^−1^.

**Conclusions:**

The sensor's high accuracy, rapid response time, and ability to selectively target tumor cells make it a breakthrough in early cancer detection. Moreover, its compact size and potential for easy fabrication enhance its applicability in emergency settings. This cutting‐edge approach represents a major advancement in medical technology, paving the way for more efficient and targeted cancer diagnostics.

## 1 Introduction

A revolutionary development in fiber optics, photonic crystal fibers (PCFs) provide unmatched structural flexibility and a wide range of uses in the telecom and nontelecommunications industries [1]. In contrast to conventional optical fibers, PCFs provide innovative and adjustable properties, such as single‐mode guiding via photonic‐band‐gap (PBG) or efficient index processes, by using special characteristics including lattice pitch, air hole geometry, and refractive index (RI) [2]. T‐rays, also known as terahertz (THz) radiation, have wavelengths between 0.1 and 10 THz and are found in the electromagnetic spectrum between radio and infrared frequencies [3, 4]. It is now a major area of study in biophysics due to its uses in safe diagnostic imaging [5], liquid infiltration analysis [6], wavelength demultiplexers [7], telecommunications [8], and glucose sensing [9]. Because of their wide communication range and capacity to facilitate electromagnetic field investigation, hollow‐core PCFs are especially preferred for THz applications. In order to evaluate fluid fluctuations in the THz spectrum, validated waveguide systems have also been created [10].

Recent developments in PCFs have significantly enhanced sensor performance for gas and pressure detection, driven by innovations like the pressure sensor utilizing water‐filled air holes and the detection of blood cancer utilizing chromatic dispersion as the primary sensing mechanism [11, 12]. Additionally, advancements in all‐optical NAND/NOR gates [13, 14] and topological edge states have paved the way for high‐speed optical logic circuits, achieving data rates of up to 5 Tb/s [15]. PCF‐based biosensors are gaining increasing attention, offering cost‐effective and flexible designs compared to other advanced technologies like nanotechnology and electronics [16, 17, 18, 19, 20, 21]. In the context of cancer detection, PCFs hold promise due to their ability to detect variations in the RI, which can identify cancerous cell concentrations quickly. Cancer, a complex genetic disorder characterized by the disruption of DNA repair processes and cellular regulation, remains one of the leading causes of death globally [22, 23, 24]. Early diagnosis is critical for successful treatment, and PCF detectors offer a novel approach to facilitating the timely identification of malignancies [25]. In 2020, Bulbul et al. proposed a sensor to identify breast cancer cells. The manufacturing capability of this sensor is demonstrated in the 92.2% RS, CL of 6.52 × 10^−14^ cm^−1^, and effective material loss (EML) of 0.0117 cm^−1^ [26]. Parvin et al. suggested a RI‐based sensor utilizing differential optical absorption spectroscopy for cancer cell detection in 2021 [27]. Shahareaj et al. with fellows suggested a highly sensitive PCF with a hybrid dodecagonal core with double‐layer hexagonal cladding for identifying health parameters in food oil in 2023 [28]. A decagonal solid‐core PCF structure for blood cell detection at a THz range is proposed by Amit et al. and achieves peak sensitivities of 84.55% for glucose, 85.09% for plasma, 85.62% for WBC, and 87.68% for RBC [29]. Yadav et al. published a research paper describing a THz sensor developed with HC‐PCF for cancer cell identification. The sensor's RS is 81.38% for cancer cells and 65.83% for normal cells with a corresponding CL of 5.828 × 10^−25^ cm^−1^ [30]. In 2023, Ibrahim et al. presented a THz PCF detector for identifying creatinine levels in blood, achieving relative sensitivities of 93% and 95% [31]. In 2024, Kundu et al. have also suggested a unique web core that can cover sensors of hexagons to determine chemical sensing performance [32]. Moreover, numerical results of this sensor show a relatively higher sensitivity of 95.21% for benzene and 94.67% for ethanol.

This study introduces and numerically evaluates a PCF‐based cancer cell detector with a circular core and curved trapezoid cladding utilizing the THz spectrum. The maximum sensitivity responses achieved are 99.60% for cervical cancer, 99.64% for adrenal gland cancer, 99.46% for skin cancer, 99.58% for blood cancer, 99.69% for breast cancer type I, and 99.71% for breast cancer type II at 2.2 THz. The research analyzes guiding properties, sensitivity response, as well as numerical aperture (NA), loss values of the sensor, effective area (EA), and spot size. Detailed discussions on the additional characteristics of this PCF‐based waveguide are provided in the results and analysis section.

## 2 Sensor Model Design

**FIGURE 1:**
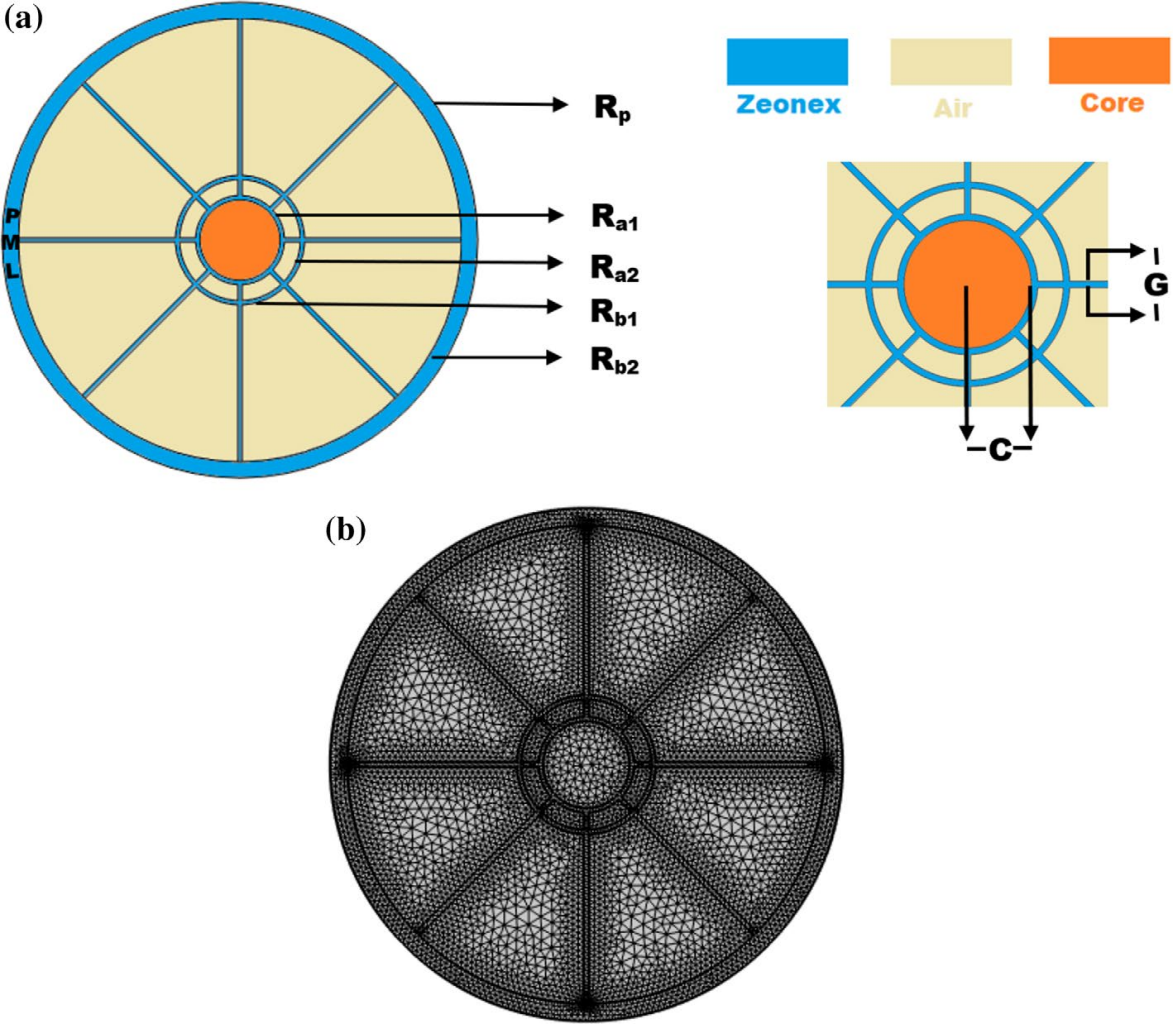
(a) Depicts the proposed PCF design's cross‐sectional aspect. (b) Showcases the mesh configuration for the intended PCF.

A proposed sensor for the detection of cancer cells using THz PCF is designed in this paper with a systematic approach based on the finite element method (FEM). It follows a sequential process: first, defining an arbitrary model of PCF, then assigning materials to the core and cladding manually or with the aid of a database. The design process emphasizes simulating the polarization‐maintaining behavior of traditional fibers. Key steps involved in the process are setting boundary conditions and mesh arrangements, solving partial differential equations, and performance evaluation in terms of sensitivity and confinement loss (CL). Value addition in cladding is introduced by two layers of curved trapezoidal air holes. Their dimensions have been optimized using a hit‐and‐trial approach in order to increase light trapping, reduce loss, and thereby maximize sensitivity. The use of materials like Zeonex with low absorption loss and high optical transparency further optimizes the performance of the sensor. A perfectly matched layer (PML) boundary condition is applied to avoid reflections from low‐refracting cladding areas. The presented FEM‐based complete methodology ensures that the design of the sensor will meet the specific requirements of THz‐based cancer cell detection with high sensitivity and minimum loss. In this process, there are several steps in sequence: defining an arbitrary PCF model automatically; assigning materials to the core and cladding region manually or from the material database for simulating polarization‐maintaining behavior of traditional fiber. etc.; setting up boundary conditions & mesh; solving partial differential equations to evaluate performance measures. These steps are analyzed and adjusted as necessary until the desired results are achieved. A sectional view of the sensor appears in Figure 1a, where both healthy and malignant cells serve as analytes.

The circular core's radius is defined as C = P. The cladding section features two layers of sixteen curved trapezoidal air holes. In the first layer, the inner hole radius is R_a1_ = 1.1P, while the exterior hole radius is R_a2_ = 1.5P. In the second layer, the inner hole radius is R_b1_ = 1.6P, while the exterior hole radius is R_b2_ = 5.5P. Two air holes are distinct by G = 0.1P. A circular PML boundary condition operates in the sensor's outermost space with a radius of R_p_ = 5.9P for preventing reflection back from lower refracting cladding areas. The pitch P varies from 100 to 190 μm, which greatly eases fabrication for the purely periodic case since only one parameter (the pitch P) is now maintained—controlling both the fiber cores and cladding holes. The proposed sensor is designed to have the benefits of enhanced light trapping, higher sensitivity, and lower loss. The trial‐and‐error approach is used to find the values for these parameters. In the realm of THz‐based PCFs, the materials that are most frequently used as a backdrop include Teflon, Zeonex, polymethyl methacrylate (PMMA), and TOPAS. These materials are widely used because of their unique characteristics as well as lower absorption loss when compared to another type of optical glasses. Out of these materials, Zeonex exhibits high optical transparency and tensile modulus coupled with the least amount of absorption losses. As a result, Zeonex is the chosen material for the background of this sensor.

The complex mesh arrangement in PCF significantly impacts its optical and sensing characteristics. Researchers can adjust the organization of ventilation pockets within the mesh to fine‐tune the fiber's acoustic behavior. Additionally, the mesh pattern directly affects PCF's transparency, influencing its ability to guide light and interact with external substances. Notably, this PCF mesh comprises 76 vertex elements, 1423 boundary elements, 15,172 total elements, and a minimum element quality of 0.4211.

**FIGURE 2:**
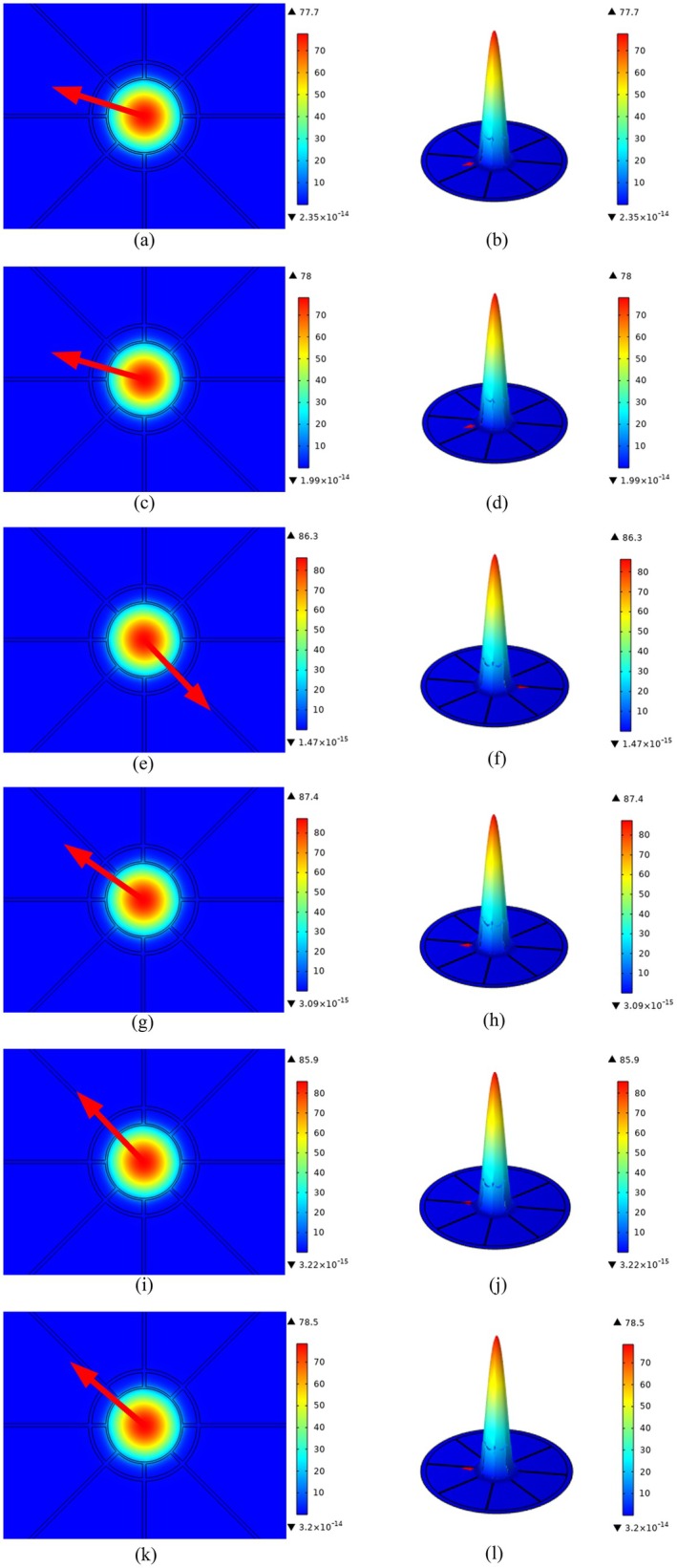
Manifest the distribution of (a) cervical cancer, (c) adrenal gland cancer, (e) skin cancer, (g) blood cancer, (i) breast cancer type I, and (k) breast cancer type II, and, power and density is hown in (b), (d), (f), (h), (j) and (l) respectively.

In Figure 2, we explore the electric power arrangement within PCF under specific conditions. Luminosity flows through the core and cladding, influenced by algebraic expressions, RI, and absorption. Researchers leverage this understanding to predict brightness changes, optimize machine performance, and detect external adjustments accurately. Density distribution characterizes material dispersion across different fiber regions, including stiffness variations. Understanding granular scattering is vital for tailoring PCFs in applications like tracking and telecommunications.

Refractive indices (RIs) of several malignant cells in comparison to normal cells are shown in Table 1 [33].

**TABLE 1:** Refractive indices of certain cancerous cells relative to their normal counterparts.

Sort of cancer	Sort of cancer cell	RI of norm cell (concentration level 30%–70%)	RI of cancer cell (concentration level 80%)
Cervical	HeLA	1.368	1.392
Adrenal glands	PC‐12	1.381	1.395
Skin	Basal	1.36	1.38
Blood	Jurkat	1.376	1.39
Breast (type 1)	MDA‐MB‐231	1.385	1.399
Breast (type 2)	MCF‐7	1.387	1.401

Each of the six cancer cell types in the dataset has a unique RI value that is important for optical sensing and comes from a different tissue. Cervical cancer is the source of HeLa cells (RI: 1.368), which are employed extensively because of their durability and quick development. Originating from a tumor of the rat adrenal gland, PC‐12 cells (RI: 1.381) are essential for studying neuroscience. Basal cell carcinoma, a prevalent kind of skin cancer, is represented by basal cells (RI: 1.36). Human T cells called Jurkat cells (RI: 1.376) are utilized to investigate immunological signaling and leukemia. Both MDA‐MB‐231 (RI: 1.385) and MCF‐7 (RI: 1.387) are cell lines used to study breast cancer; the former is triple‐negative and aggressive, while the latter is hormone‐receptor‐positive and frequently utilized in hormonal treatment research.

## 3 Results

Our investigations rely on extensive simulations utilizing the vector FEM in conjunction with COMSOL Multiphysics V6.1a software. This crafted technique effectively addresses the numeric solution of partial differential equations in both two and three dimensions, considering not only temporal factors but also spatial dependencies. We evaluate the refraction properties of each constituent by embedding geometrical features and RIs. FEM can be used to quantitatively solve these equations, giving the detailed information of light propagation in PCF, as well as its distribution and acoustic properties.

RS is regarded as one of a sensor's most crucial characteristics since it indicates how much of an element has the specific elements that are being detected. Typically, this sensing endeavor involves aligning with the RI for precision. The computation of light intensity interacting directly with the detecting analyte serves as a pivotal step in establishing RS [34]. The RS of analytes can be mathematically measured using the expression (1) [35].

(1) $$r=\frac{n_{r}}{n_{\mathrm{eff}}}\times p\%$$

In this case, *n*_*r*_ symbolizes analyte's RI and *n*_eff_ symbolizes effective state RI. Furthermore, *p* indicative of power rate facilitates measuring the light bound to sensor materials within the core section. It can be assessed using dthe subsequent Equation (2) [35].

(2) $$p=\frac{\int_{\text{sample}}R_{e}\left(E_{x}H_{y}-E_{y}H_{x}\right)\text{dxdy}}{\int_{\text{total}}R_{e}\left(E_{x}H_{y}-E_{y}H_{x}\right)\text{dxdy}}$$

The evaluation entails delineating light propagation across the fiber's core region alongside its overall cross‐sectional area by illustrating the numerator and denominator, respectively. Specifically, components of magnetic and electric fields are defined as *E*_*x*_, *E*_*y*_, and *H*_*x*_, *H*_*y*_, whereas directions of polarization mode are distinguished through subscripts *x* and *y*.

**FIGURE 3:**
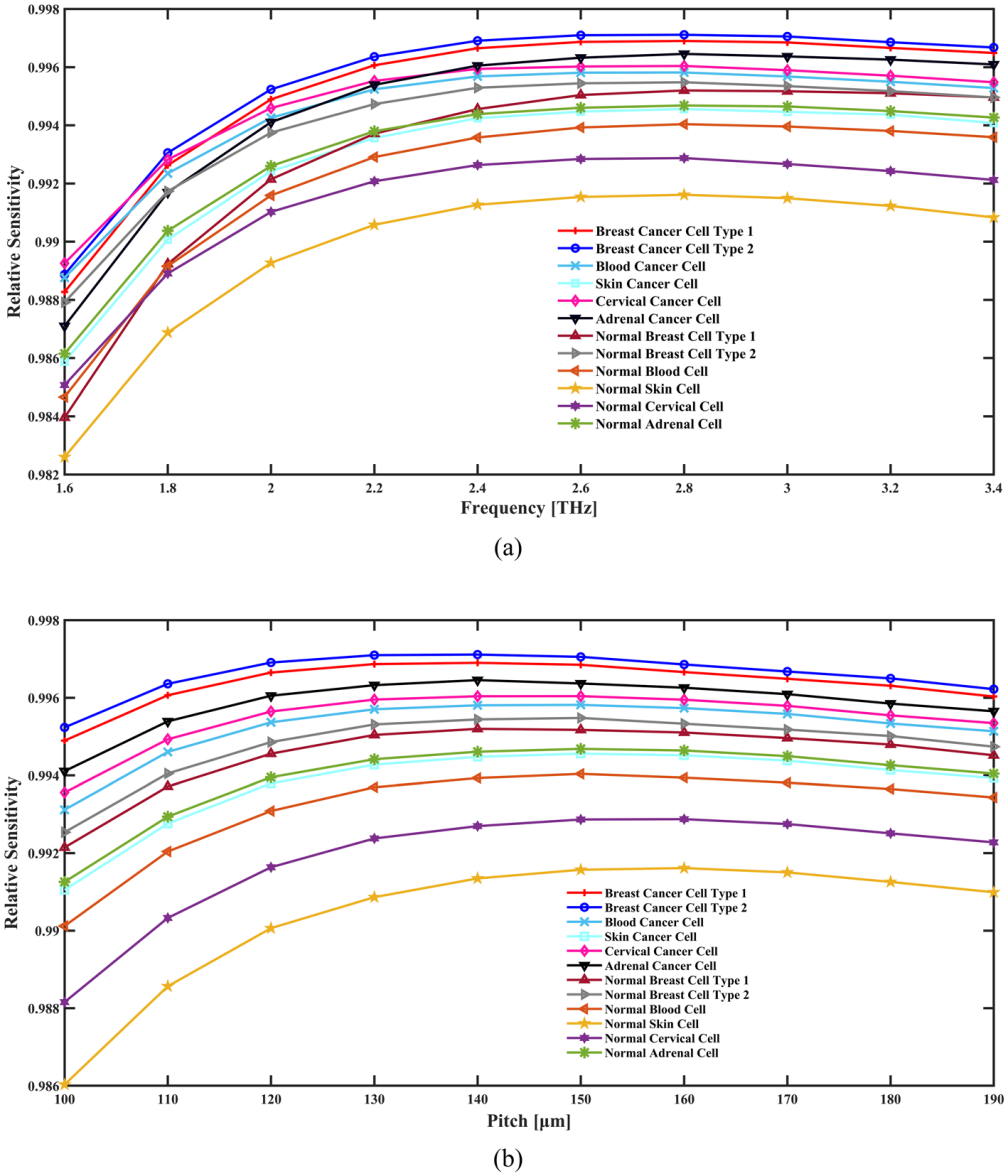
Indicate the effects of the RS in terms of (a) frequency [THz] and (b) pitch [μm].

With the increase in pitch and frequency, the THz PCF sensor, because of better light‐matter interaction and mode confinement, becomes more relatively sensitive. The spacing between the air holes in the cladding increases with increasing pitch and hence decreases the effective RI of the cladding. This serves to extend more of the guided optical field into the core where the analyte—such as cancer cells—is located. This interaction enhances the sensitivity through allowing the optical mode to better “sense” changes in the RI of the analyte. Similarly, as the frequency increases, the wavelength becomes shorter, leading to tighter confinement of the optical mode in the core. This tighter confinement increases the overlap between the guided light and the analyte, further enhancing sensitivity. Both factors contribute synergistically to improving the sensor's RS, making it more effective for detecting subtle changes in the RI caused by cancer cells. These parameters have to be balanced to optimize the performance of the sensor for clinical applications. An elaborate illustration showcasing sensitivity assessment across diverse frequencies and geometrical parameters is vividly portrayed in Figure 3a,b. Noteworthy is the RS performance curve concerning sensor operation within a frequency spectrum spanning 1.6 to 3.4 THz depicted in Figure 3a. Evidently, the proposed fiber exhibits peak RS at 2.8 THz amidst varied core radius fitting between 100 and 190 μm overseeing optimal PCF‐based cancer cell detection which is shown in Figure 3b. The figure illustrates that under optimal circumstances, the proposed sensor exhibits relative sensitivities of 99.60% for cervical cancer, 99.64% for adrenal gland cancer, 99.46% for skin cancer, 99.58% for blood cancer, 99.69% for breast cancer type I, and 99.71% for breast cancer type II. Additionally, when these cells are in a normal state, the relative sensitivities are 99.28%, 99.47%, 99.16%, 99.40%, 99.52%, and 99.55%, respectively. It is crucial to note that in real‐world applications, the suggested fiber's sensitivity cannot be higher than 100%.

Light‐gathering prowess and angle acceptance of a PCF find their expression in the NA. A greater NA signifies a broader range of light collection orientations, akin to opening more windows for photons to enter. These factors impact how light interacts with the PCF, affecting its ability to guide and transmit photons. Understanding and fine‐tuning NA is essential. It empowers one to meet the diverse demands of optical systems and components. Use the following mathematical formula to determine NA [36].

(3) $$\mathrm{NA}=\frac{1}{\sqrt{1+\frac{{\mathrm{\pi A}}_{\mathrm{eff}}f^{2}}{c^{2}}}}\approx \frac{1}{\sqrt{1+\frac{{\mathrm{\pi A}}_{\mathrm{eff}}}{\lambda^{2}}}}$$

**FIGURE 4:**
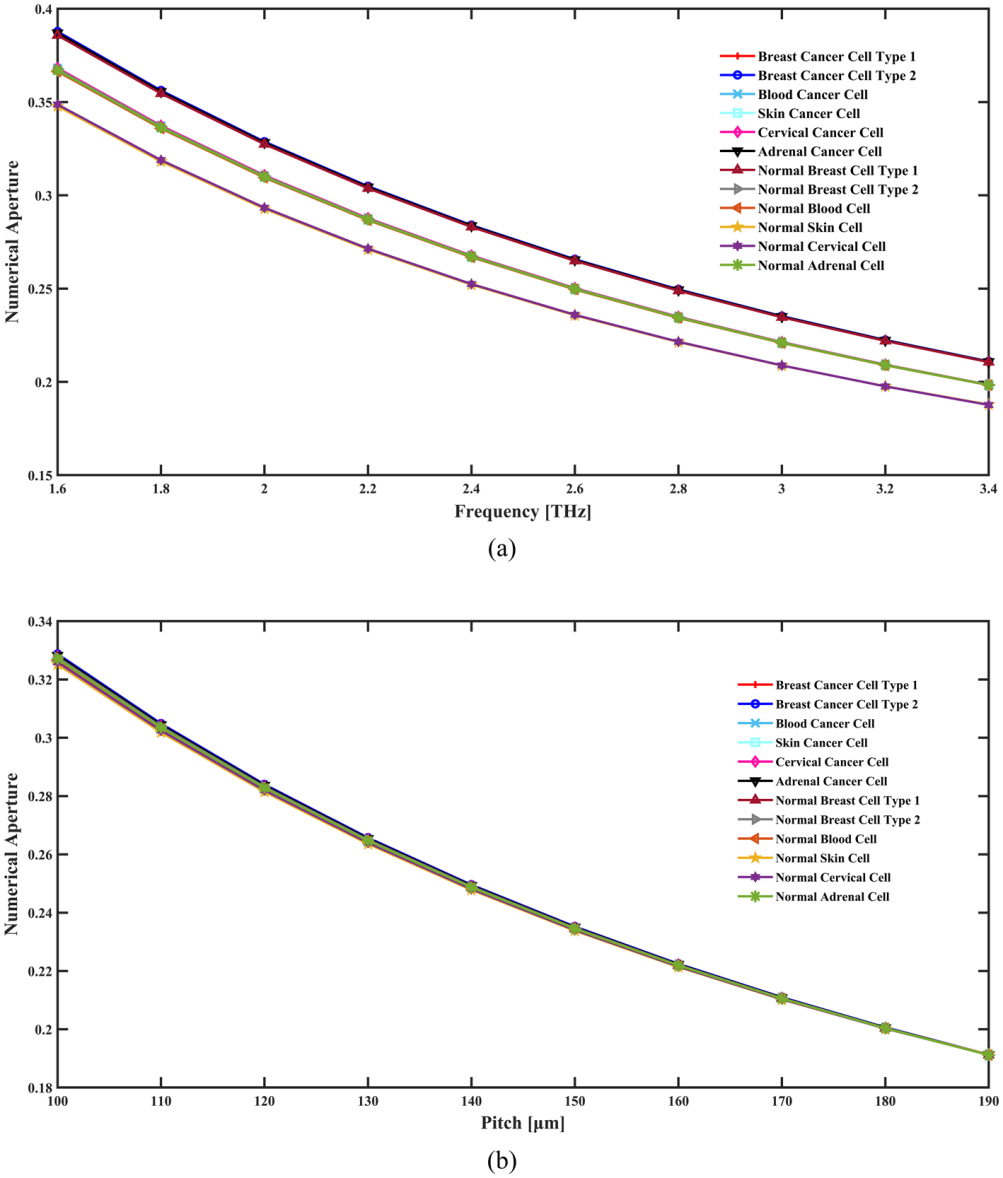
Indicate the effects of NA in terms of (a) frequency [THz] and (b) pitch [μm].

Here, the open space light velocity is denoted as *c*, the wavelength as *λ* and *A*_eff_ corresponds to the effective signal propagating area. The given sensor offers a favorable NA of around 0.235 for cervical cancer cells, 0.249 for adrenal gland cancer cells, 0.234 for skin cancer cells, 0.235 for blood cancer cells, 0.249 for breast cancer type I cells, and 0.249 for breast cancer type II cell detection at 2.8 THz under ideal geometric conditions. When these cells are in a normal state, NA are 0.222, 0.234, 0.221, 0.234, 0.248, and 0.235, respectively. The variance in NA across several operating frequency ranges is seen in Figure 4a. In contrast, Figure 4b shows how NA varies in response to pitch variations.

The EML takes into consideration all of the many loss processes that are present in the fiber structure. As light travels through the PCF, it undergoes several processes such as dispersion, absorption, and other elements that lower its mean intensity. PCF‐based photonic systems are evaluated using EML as a crucial parameter since it has a substantial influence on signal transmission and system dependability. Use the following mathematical formula to determine EML [10].

(4) $$\alpha_{\mathrm{eff}}=\frac{{\left(\frac{\varepsilon_{0}}{\mu_{0}}\right)}^{\frac{1}{2}}\;\int_{A_{\max}}n\alpha_{\mathrm{mat}}{\left|E\right|}^{2}\mathrm{dA}}{2\int_{\mathrm{ALL}}S_{z}\mathrm{dA}}$$

Here, absorption loss of overall material and Zeonex's RI are represented by *α*_mat_ and *η*, respectively. Delineating the Poynting vector's *z*‐component recognized as *S*_*z*_ = ½ × (*E* × *H*), in which *E* and *H*, respectively, represent the complicated form magnetic field and electric field ingredient alongside $\varepsilon_{0}$ and $\mu_{0}$ representing permittivity and permeability within free space.

**FIGURE 5:**
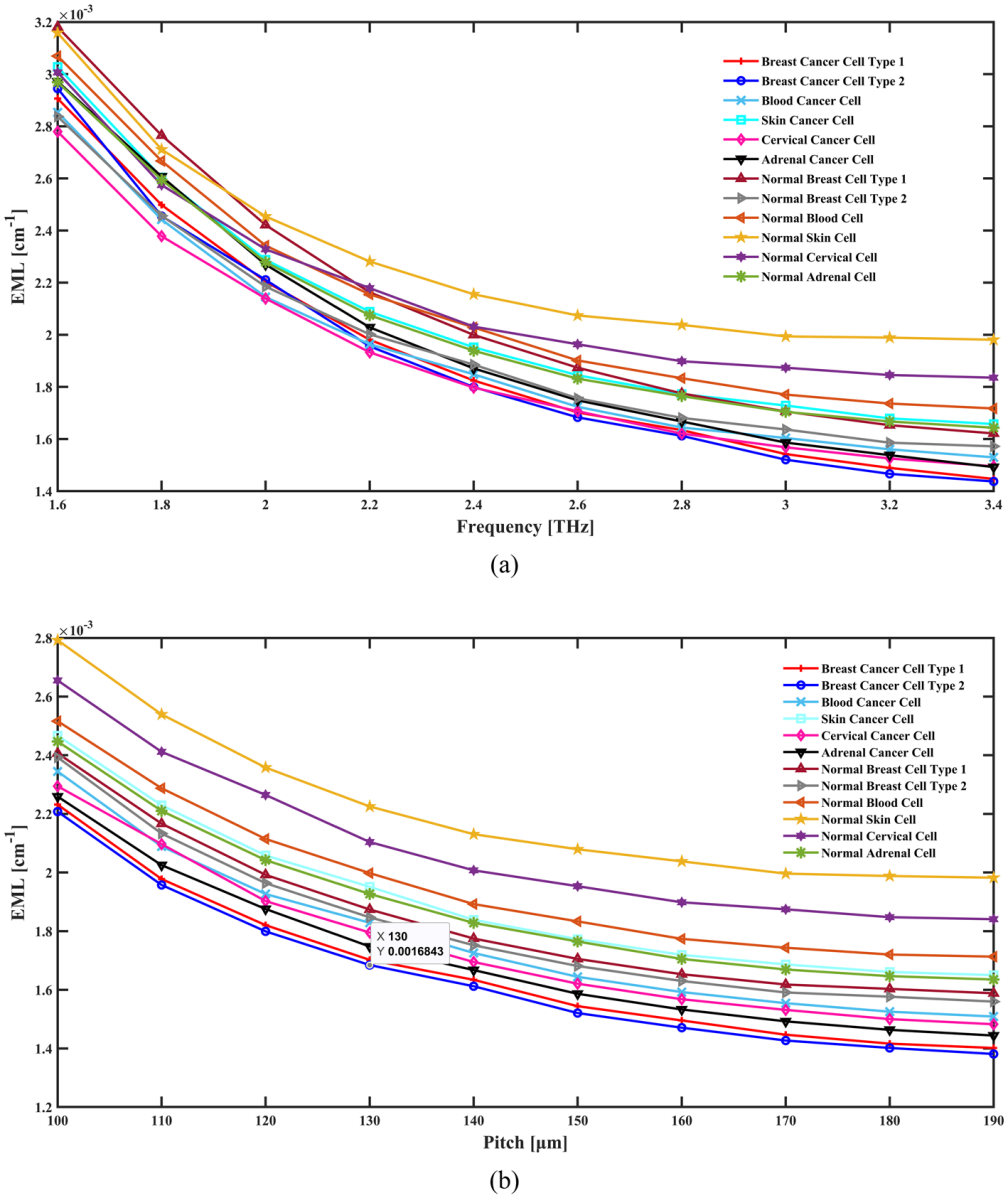
Indicate the effects of the EML in terms of (a) frequency [THz] and (b) pitch [μm].

The EML of this presented sensor is about 0.00162 cm^−1^ for cervical cancer cells, 0.00167 cm^−1^ for adrenal gland cancer cells, 0.00177 cm^−1^ for skin cancer cells, 0.00164 cm^−1^ for blood cancer cells, 0.00163 cm^−1^ for breast cancer type I cells, and 0.00161 cm^−1^ for breast cancer type II cells. When these cells are in a normal state, the EML is 0.00189, 0.00176, 0.00204, 0.00183, 0.00177 and 0.00168 cm,^−1^ respectively. The variance in EML across several operating frequency ranges is seen in Figure 5a. A decrease in light propagation as a result of the cancer cells changing optical characteristics is suggested by the observed drop in EML values for cancer cells when compared to their normal counterparts. This pattern demonstrates how sensitive the PCF sensor is to cellular alterations. In contrast, Figure 5b shows how EML varies in response to pitch variations. A higher pitch decreases material loss, as seen by the fluctuation of EML with pitch variations. This is probably because of better mode confinement and less scattering in the PCF.

In PCF, CL is a further category of inevitable loss. CL in a PCF occurs when light escapes from the center to the coating. Reducing this loss is essential to ensure that transmitted information remains within the core, thereby avoiding dispersion. Calculation of the CL involves factors such as the air hole diameter, pitch, and shape of the PCF. Use the following mathematical formula to determine CL [37].

(5) $$\alpha_{\mathrm{CL}}=8.686\left(\frac{2\mathrm{\pi f}}{c}\right)\mathrm{Img}\left(n_{\mathrm{eff}}\right)$$

**FIGURE 6:**
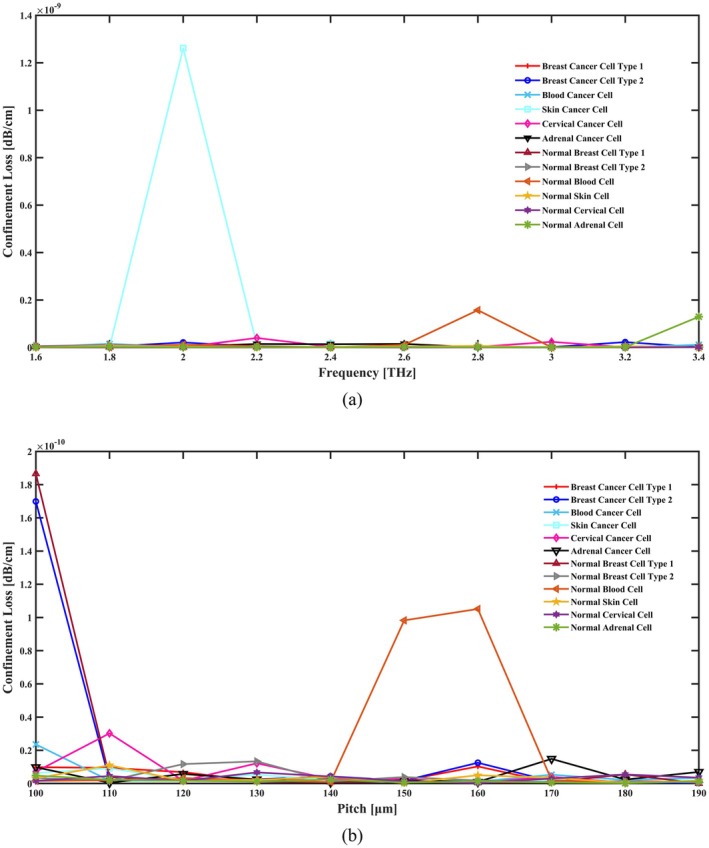
Indicate the effects of the CL in terms of (a) frequency [THz] and (b) pitch [μm].

At which *c* is the light wave's free space mobility, *f* stands for frequency, and Img(neff) represents the imaginary component of RI. The CL of our suggested detecting system for different frequency and pitch changes is displayed in Figure 6. The lower CL values for cancer cells compared to normal cells in Figure 6a suggest more effective light confinement, most likely as a result of RI alterations. The interaction between guided light and the fiber's material characteristics causes the variations in CL across frequencies. The fluctuation in CL may be explained by increased losses caused by an increase in the imaginary component of the RI at higher frequencies. In Figure 6b, CL changes with pitch due to its effect on light confinement in the PCF. Larger pitches decrease CL by enhancing confinement inside the core, while smaller pitches increase CL by allowing more light to escape from the core. This variation demonstrates how pitch changes affect the efficiency of signal transmission and light leakage.

According to the numerical results, the CL of this sensor is 2.61 × 10^−12^ dB/cm for cervical cancer cells, 6.69 × 10^−13^ dB/cm for adrenal gland cancer cells, 1.57 × 10^−12^ dB/cm for skin cancer cells, 1.29 × 10^−12^ dB/cm for blood cancer cells, 2.80 × 10^−12^ dB/cm for breast cancer type I cells, and 3.39 × 10^−12^ dB/cm for breast cancer type II cell detection at 2.8 THz under ideal geometric conditions. When these cells are in a normal state, the CL is 3.23 × 10^−13^ dB/cm, 1.26 × 10^−12^ dB/cm, 6.45 × 10^−12^ dB/cm, 1.57 × 10^−10^ dB/cm, 8.82 × 10^−13^ dB/cm, and 5.16 × 10^−12^ dB/cm, respectively.

**FIGURE 7:**
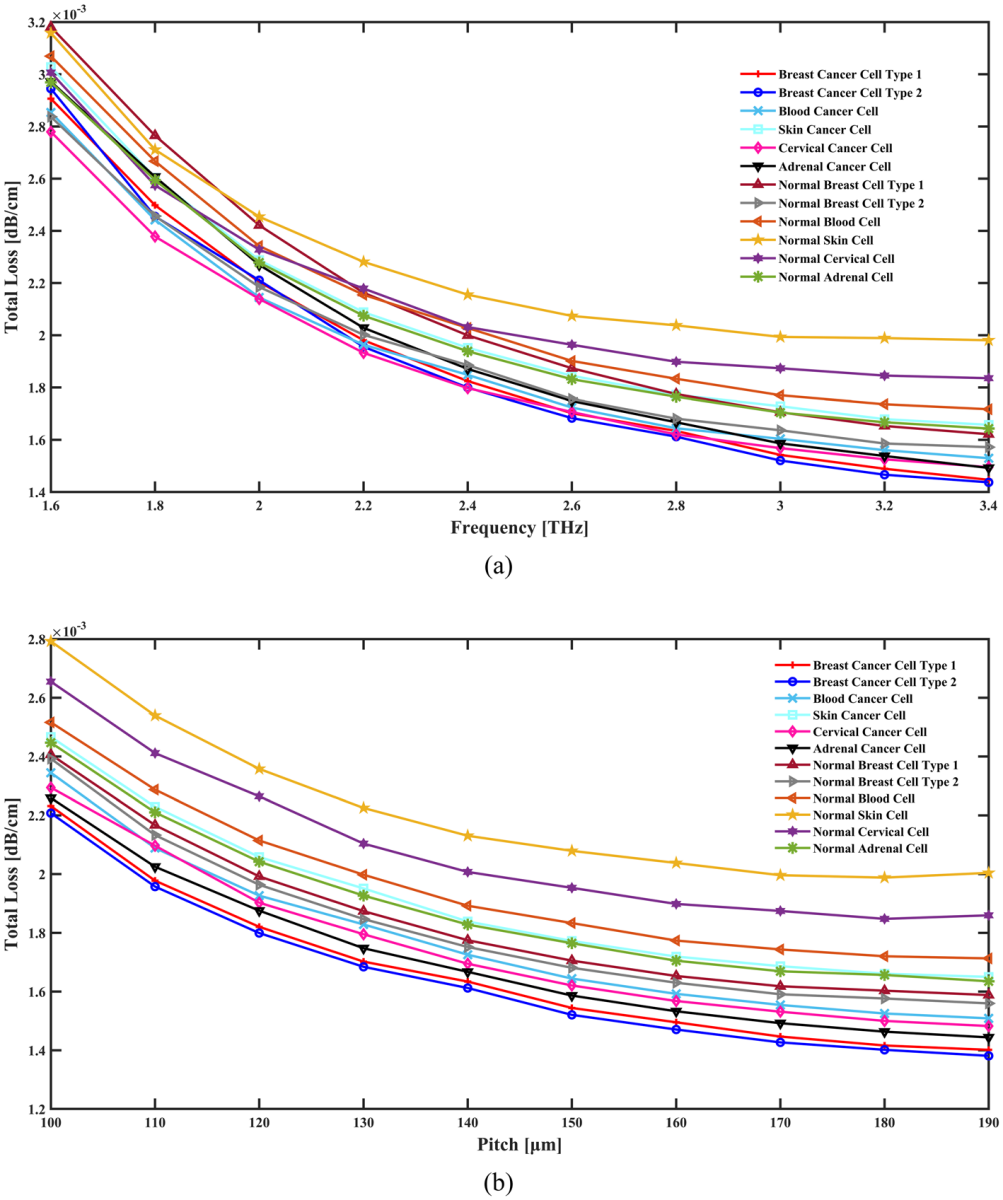
Indicate the effects of the total loss in terms of (a) frequency [THz] and (b) pitch [μm].

**FIGURE 8:**
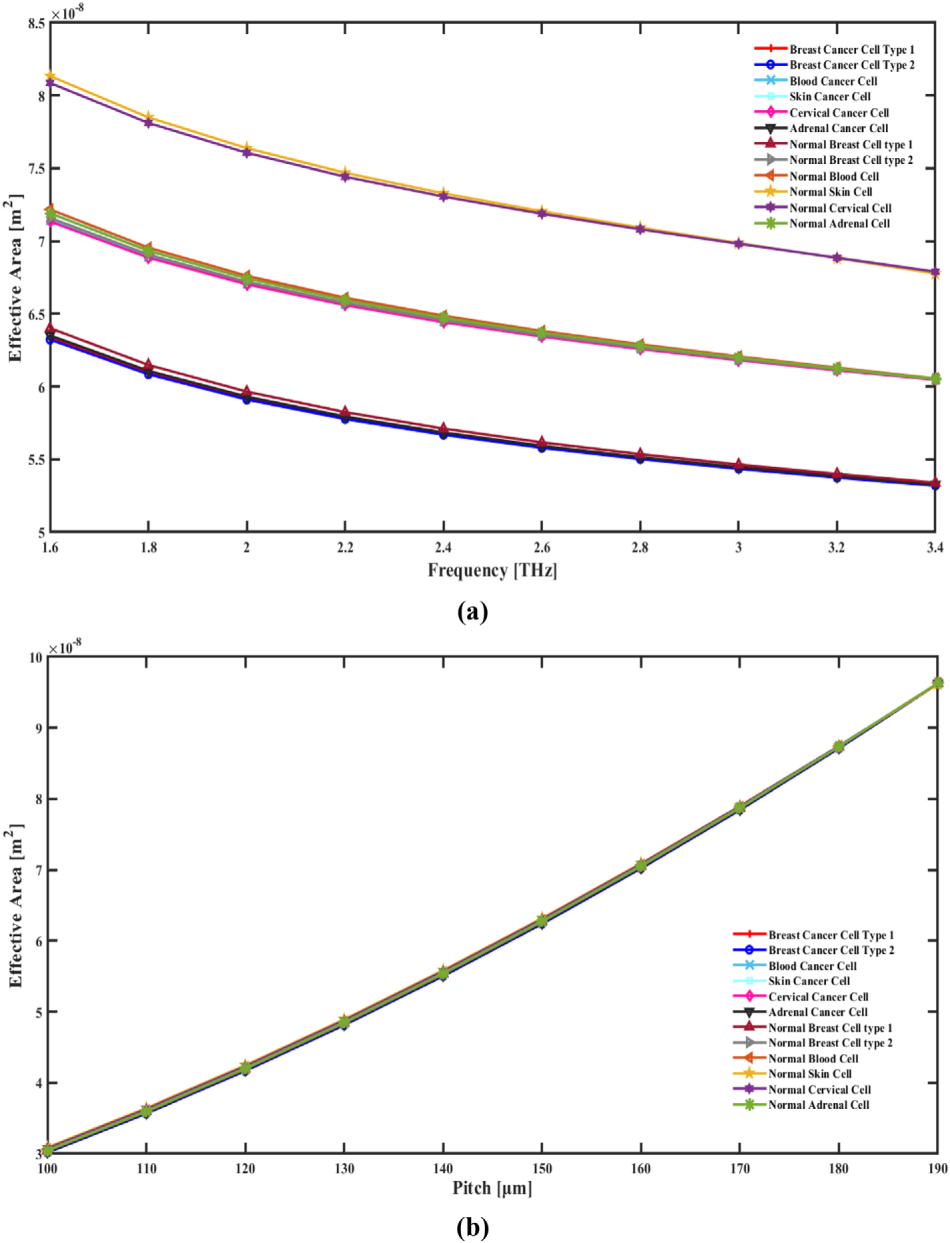
Indicate the effects of the EA in terms of (a) frequency [THz] and (b) pitch [μm].

EML plus CL add up to the total loss of this proposed cancer cell detecting sensor, which is shown in Figure 7 for various operating frequencies and geometrical parameters. The main reason for the reduction in total loss within the THz photonic crystal fiber (PCF) Sensor for Cancer Cell Detection is the increase in frequency and pitch, which is illustrated in Figure 8a,b because of better confinement of THz waves within the core of the fiber. With increasing pitch, which is illustrated in Figure 8a (distance between the air holes in the cladding), there is a more effective spacing between these structures that leads to the formation of a photonic bandgap, thereby reducing the leakage of light to the cladding region. This results in low CL, which is actually the most dominant loss mechanism in PCFs. Secondly, at higher frequencies, the effective interaction between the guided THz wave and the cladding periodic air hole structure becomes much stronger. The higher frequency will improve the fiber's capability to suppress unwanted modes and maintain the desired modes of propagation in the core, further reducing scattering and absorption losses. These factors combined guarantee improved light guidance and overall lower optical losses, which means higher sensitivity and accuracy in the detection of cancer cells by the sensor. The total losses are about 1.621 × 10^−03^ dB/cm for cervical cancer cell, 1.667 × 10^−03^ dB/cm for adrenal gland cancer cell, 1.772 × 10^−03^ dB/cm for skin cancer cell, 1.644 × 10^−03^ dB/cm for blood cancer cell, 1.634 × 10^−03^ dB/cm for breast cancer type I cell, and 1.612 × 10^−03^ dB/cm for breast cancer type II cell detection. When these cells are in a normal state, the total losses are 1.898 × 10^−03^ dB/cm, 1.765 × 10^−03^ dB/cm, 2.038 × 10^−03^ dB/cm, 1.833 × 10^−03^ dB/cm, 1.775 × 10^−03^ dB/cm, and 1.681 × 10^−03^ dB/cm, respectively. A steep decline in the overall loss as a function of the initial rise in frequencies is seen in Figure 7a. Higher core diameters also result in total loss reductions, as seen in Figure 7b.

Within a fiber, the EA measures its geographical spread of coherent mode propagation. It affects the interaction between light and the fiber and is defined by fiber geometry as well as the pattern of RIs. The EA is a function of various factors such as optoelectronic spectrum gaps framework, core dimensions, and the difference between RIs (between cladding and core). A smaller EA indicates a more confined mode, while a larger EA suggests a more spread‐out state. Controlling the EA allows for the customization of PCF designs to enhance interactions between light and the medium, thereby improving performance in signal communication and nonlinear optical applications. The EA is calculated via Equation (6) [38].

(6) $$A_{\mathrm{eff}}=\frac{{\left[\int I\left(r\right)\mathrm{rdr}\right]}^{2}}{{\left[\int I^{2}\left(r\right)\mathrm{rdr}\right]}^{2}}$$

where *I*(*r*) = |*E*_*t*_|^2^ stands for the electrical field strength optical sensor, while *A*_eff_ refers to EA.

In the THz PCF Sensor for Cancer Cell Detection, the EA decreases with an increase in frequency, which is illustrated in Figure 8a due to the stronger confinement of the electromagnetic field within the core region at higher frequencies. As frequency increases, the wavelength of the THz wave shortens; hence, the optical mode becomes localized tighter. This smaller spread of the mode field corresponds to a reduced EA, which increases the sensitivity by strengthening the light‐matter interaction within the core. On the other hand, the EA increases by increasing the pitch, which is illustrated in Figure 8b, understood as the distance between air holes in the cladding. An increased pitch means a less dense cladding structure and thus gives more freedom for the mode field to expand into the surrounding cladding region. This expansion increases the EA and reduces the power density in the core, but simultaneously lowers CL. The balance between pitch and frequency allows for a very precise engineering of the PCF design to realize an optimum performance for certain sensing applications. The EA of this sensor is 6.257 × 10^−08^ m^2^ for cervical cancer cells, 5.514 × 10^−08^ m^2^ for adrenal gland cancer cells, 6.282 × 10^−08^ m^2^ for skin cancer cells, 6.262 × 10^−08^ m^2^ for blood cancer cells, 5.506 × 10^−08^ m^2^ for breast cancer type I cells, and 5.502 × 10^−08^ m^2^ for breast cancer type II cell detection. Additionally, when these cells are in a normal state, the EAs are 7.081 × 10^−08^ m^2^, 6.279 × 10^−08^ m^2^, 7.092 × 10^−08^ m^2^, 6.290 × 10^−08^ m^2^, 5.535 × 10^−08^ m^2^, and 6.268 × 10^−08^ m^2^, respectively. Figure 8 reveals the EA of our proposed detecting sensor at different frequency and pitch changes.

The coherent pattern that propagates through the fiber core usually has a physical image size, and this is known as the spot size in PCF. The viewable area's geographic extent is delineated, along with the electromagnetic radiation concentration and center of gravity. The wavelength of light, the fiber's shape, and the RI pattern are some factors that affect spot size. In addition, the factor that affects the spot size in PCF is its photonic‐band‐gap layout and RI difference between cladding and core. Smaller spot sizes offer tighter confinement and greater spatial resolution, whereas larger spot sizes suggest more light dispersion.

**FIGURE 9:**
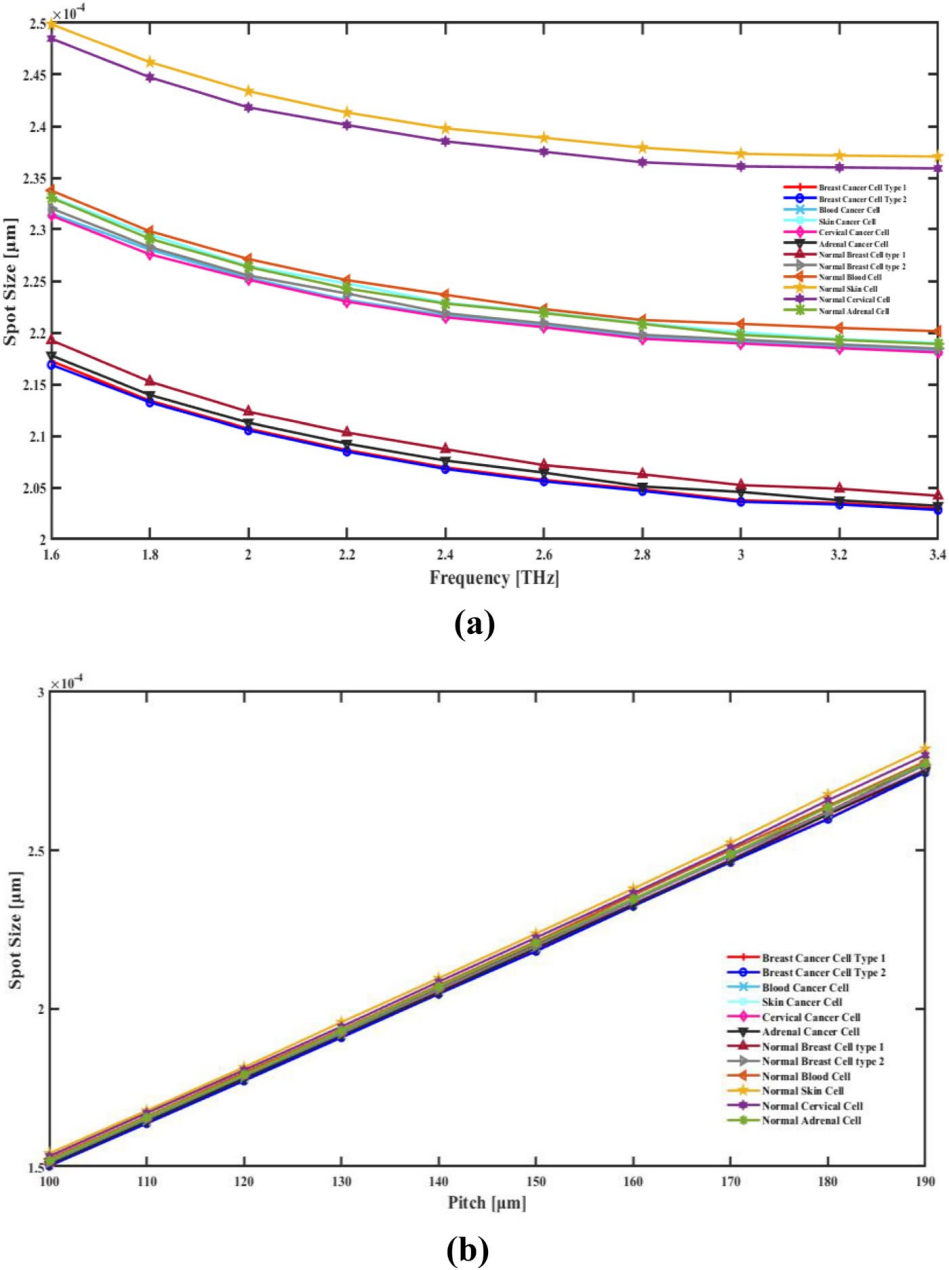
Indicate the effects of the spot size in terms of (a) frequency [THz] and (b) pitch [μm].

Use the following mathematical formula to determine spot size [39].

(7) $$W_{\mathrm{eff}}=R\times \left(0.65\times 1.619\times V^{-1.5}+2.879\times V^{-6}\right)$$

where *V* is the frequency parameter value and *R* denotes the core radius. With the increase in frequency in Figure 9a, the spot size of a PCF decreases because higher frequencies mean shorter wavelengths. As the wavelength becomes smaller, the optical mode becomes more tightly confined within the core, reducing the spot size. It is this stronger confinement that enhances the interaction of the sensor with the analytes in the core region, therefore enhancing sensitivity and precision in the detection of cancer cells. On the other hand, the spot size increases with an increase in pitch, which is shown in Figure 9b, which is the distance between the air holes in the cladding. A larger pitch reduces the RI contrast between the core and cladding, allowing the optical mode to spread more into the cladding region. In consequence of a larger mode field, spot size increases, reducing possibly the intensity of the light in the core while offering the advantage of improving guiding stability, reduction of CL, thus requesting a careful adjustment of the pitch and frequency in balance with such effects for optimizing performance that these sensors can deliver for detecting cancerous cells. The spot sizes of this sensor are 2.194 × 10^−04^ μm for cervical cancer, 2.051 × 10^−04^ μm for adrenal gland cancer, 2.209 × 10 μm^−04^ for skin cancer μm, 2.196 × 10^−04^ μm for blood cancer, 2.048 × 10^−04^ μm for breast cancer type I, and 2.047 × 10^−04^ μm for breast cancer type II cell detection. When these cancer cells are in a normal state, the sensor exhibits the spot sizes as 2.365 × 10^−04^ μm, 2.208 × 10^−04^ μm, 2.379 × 10^−04^ μm, 2.212 × 10^−04^ μm, 2.063 × 10^−04^ μm and 2.198 × 10^−04^ μm, respectively. Figure 9 illustrates the correlation between pitch, frequency, and spot size.

The response times of the proposed THz PCF sensor in experiments varied with different types of cancer cells, showing fast detection capabilities within milliseconds due to the efficient interaction of THz waves with cellular RIs. It possesses a high degree of sensitivity, compact size, and speed in response, hence a good alternative for the early detection of cancers by non‐invasive means as compared to existing cancer detection technologies. Sensitivity is highly enhanced with an optimized cladding and core design to minimize optical loss and maximize light confinement, which is incomparable to conventional fiber‐based sensors. While the design and simulations highlight its theoretical effectiveness, real‐world tests in emergency scenarios or clinical settings remain to be fully conducted. Confirmation of its practicality will need further experiments with diverse cancer cell samples under real conditions to validate the performance regarding speed, sensitivity, and reliability. Full realization of the sensor's capability for integration into portable diagnostic tools would even more substantially enhance its potential application in emergency and point‐of‐care uses, bridging the gap between theoretical design and clinical implementation.

The requirement of machine learning approaches for design and optimization of this sensor is based on the system's performance parameter complexity as high, and precise discrimination of the cancer cells being needed. Large sets of data developed while designing the sensor, for instance, geometrical configurations, material properties, and factors such as sensitivity and CL of the sensor's performance, can be processed with machine learning. Through examination of these datasets, machine learning algorithms can determine optimal settings much faster and more precisely than classical trial‐and‐error procedures. Machine learning can also optimize the discrimination capacity of the sensor by mapping slight differences in THz wave interactions with cancerous and healthy cells, allowing for extremely sensitive and specific detection. This method not only speeds up the design process but also enhances sensor performance, and it is an important tool for furthering THz PCF technology in medical diagnosis [40]. Random Forest Regressor is employed to predict various parameters with changing frequency. In this research, we employed an unweighted Random Forest Regressor to predict the correlation between frequency (THz) and other aforementioned properties. The unweighted strategy guarantees the proper handling of all the data samples in balance during training since the dataset was balanced and no explicit prioritization of samples was needed. The Random Forest Regressor algorithm was employed utilizing the scikit‐learn algorithm with a number of estimators 100 and the random state at 42.

Mean square error quantifies the mean squared deviation between actual ($y_{i}$) and predicted (${\hat{y}}_{i}$) values:

(8) $$\mathrm{MSE}=\frac{1}{n}\sum_{i=1}^{n}{\left(y_{i}-{\hat{y}}_{i}\right)}^{2}$$

*R*‐squared (*R* ^2^) quantifies the fraction of variance in the dependent variable that can be anticipated from the independent variable. The range is from 0 to 1:

(9) $$R^{2}=1-\frac{\sum_{i=1}^{n}{\left(y_{i}-{\hat{y}}_{i}\right)}^{2}}{\sum_{i=1}^{n}{\left(y_{i}-{\bar{y}}_{i}\right)}^{2}}$$

where, ${\bar{y}}_{i}$ is the mean of the actual value.

**FIGURE 10:**
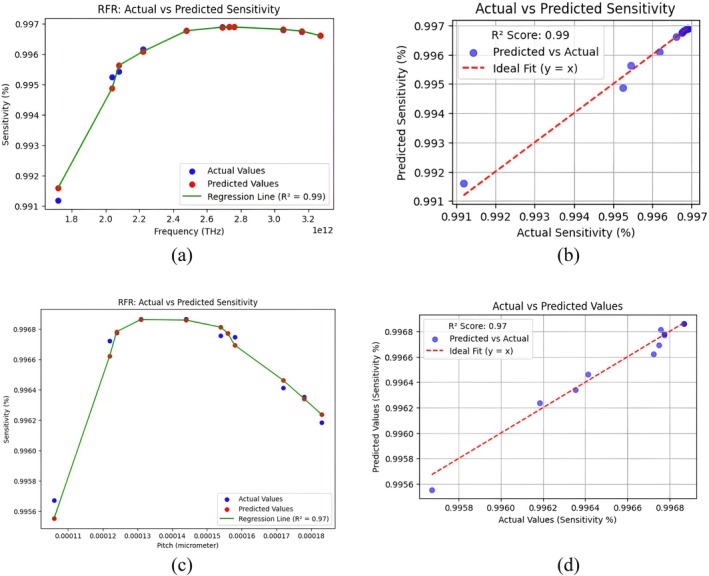
Actual versus predicted value of sensitivity is shown in (a,b) by varying frequency and in (c,d) by varying pitch.

The prediction plot generated by this algorithm is illustrated in Figure 10 for this sensor where RS *R*‐squared (*R* ^2^) score is 0.97409 and mean square error is 3.23066 × 10^−09^ for pitch variation. On the other hand, RS *R*‐squared (*R* ^2^) score is 0.98813, and the mean square error is 3.0867 × 10^−08^ for frequency variation.

Table 2 evaluates the proposed PCF model against other PCF designs that have been published based on multiple performance criteria, showing that the new model offers better sensitivity than the earlier designs.

**TABLE 2:** Compares the characteristics of the device currently in use with the attributes of the recommended analyzer to determine which works better.

Ref.	Structure	Freq. (THz)	Sample	RS (%)	CL (dB/cm)	EML (cm^−1^)
[41]	Rotating‐hexa core heptagonal cladding	1.0	Ethanol	68.48	2.13 × 10^−9^	—
			Benzene	69.20	1.92 × 10^−9^	—
			Water	66.78	2.70 × 10^−9^	—
[42]	Core and cladding rectangular‐shaped	1.8	RBCs	94.38	2.75 × 10^−13^	—
			Hemoglobin	93.72	2.87 × 10^−13^	—
			WBCs	92.94	3.06 × 10^−13^	—
			Plasma	92.14	3.27 × 10^−13^	—
			Water	90.8	3.48 × 10^−13^	—
[43]	Rectangular core	2.0	Blood cancer	96.74	2.41 × 10^−14^	0.01131
			Skin cancer	96.61	1.58 × 10^−15^	0.01131
[30]	Mono‐rectangular	2.1	Cancer	81.38	5.82 × 10^−25^	—
			Normal	65.83	3.07 × 10^−27^	—
[27]	Compact cladding with a circular lattice structure of air gaps	Wavelength 1.4–2.5 μm	Cervical cancer	94.96	5.3 × 10^−9^	—
			Adrenal gland cancer	95.15	4.3 × 10^−9^	—
			Skin cancer	94.13	3.6 × 10^−8^	—
			Blood cancer	94.84	6.1 × 10^−9^	—
			Breast cancer type‐1	95.40	5.3 × 10^−9^	—
			Breast cancer type‐1	95.51	7.5 × 10^−9^	
[44]	Circular Core	2.5	Cervical cancer	98.12	10^−8^	5.5 × 10^−3^
			Adrenal gland cancer	98.21		
			Skin cancer	97.95		
			Blood cancer	98.40		
			Breast cancer type‐1	98.34		
			Breast cancer type‐2	98.40		
This PCF	Circular core with curved trapezoidal shaped cladding	2.8	Cervical cancer	99.60	2.61 × 10^−12^	0.00162
			Adrenal gland cancer	99.64	6.69 × 10^−13^	0.00167
			Skin cancer	99.46	1.57 × 10^−12^	0.00177
			Blood cancer	99.58	1.29 × 10^−12^	0.00164
			Breast cancer type‐1	99.69	2.80 × 10^−12^	0.00163
			Breast cancer type‐2	99.71	3.39 × 10^−12^	0.00161

We held a formal discussion regarding our proposed sensor and the existing PCF monitoring system. To complement our PCF results, Table 2 outlines the key components of the current PCF. The proposed THz PCF sensor is very scalable and economical for cancer cell sensing. The design makes extensive use of readily available materials like Zeonex, which is highly optically transparent with low absorption loss, to make it low‐cost fabrication without performance compromise. The pitch‐reduced periodic structure and hit‐and‐trial parameter tunability reduce manufacturing complexity, allowing reproducibility in small lots with minimal tuning. Expanded manufacturing techniques such as stack‐and‐draw, laser‐etched, or 3D printing can also minimize the process of production without losing precise geometries. Additionally, opportunities for miniaturization and use in microfluidics make it more viable to be miniaturized for handheld diagnostic devices at a reasonable cost to facilitate further popularity in clinical and research settings. The suggested biosensor, featuring a straightforward design, exterior sensing mechanism as well as plasmonic material coating, shows improved sensing parameters compared to existing sensors. It can accurately differentiate between various malignant and healthy cells, making it useful for biomedical applications in cancer detection.

## 4 Fabrication Feasibilities and Discussion

The fabrication challenges in the proposed design mainly involved the creation of an accurate and stable structure for the PCF with curved trapezoidal air holes in the cladding. The periodic nature of the design, defined by the pitch (P), simplifies fabrication by reducing the need for complex multi‐parameter adjustments, enabling consistent replication of the intricate geometrical structure. This led to the choice of Zeonex as the background material, resolving issues with absorption losses and optical transparency and providing a good balance of mechanical stability and low attenuation in the THz regime. The curved trapezoidal air holes in the cladding contribute to an increased confinement of light and, at the same time, reduce optical losses, while the well‐defined core radius C = P ensures efficient trapping of light within the sensor. With a multi‐layer cladding design (with varying air hole radii and spacing), the strong RI contrast enhances the sensitivity and specificity of the sensor by allowing for better interaction between guided THz waves and cancer cell analytes. The FEM simulations allow for fine‐tuning of mesh configurations and boundary conditions for high‐performance sensors. The combination of these innovative cladding and core designs greatly enhances the discrimination capability of the sensor between healthy and malignant cells with high precision. Existing techniques for the creation of PCFs combine a variety of technologies, such as stacking [45] and sol–gel production methods [46] and 3D printing [47] or extraction [48]. Drilling and slurry casting enable fabrication at sub‐micron scales, among other things, as specified in certain sources. Experimental implementation of the proposed THz PCF sensor would be viable; however, it needs detailed engineering and sophisticated fabrication techniques for a trade‐off between optical performance, mechanical strength, and application requirements. The use of Zeonex as the background material will make the fabrication process easier due to its properties of high optical transparency and low absorption loss in the THz regime, ensuring better light guidance. The curved trapezoidal air holes and layered cladding structure may be fabricated by adapting appropriate fabrication processes for accurate geometry, such as stack‐and‐draw techniques, laser‐assisted etching, or 3D microprinting. For maintaining consistent optical performance, the dimensions of the air holes and their pitches (P) should be uniform; this can be realized by advanced lithography and scrupulous quality control. The experimental verification of the sensor design relies on the accurate setup of the PML boundary condition to avoid reflections and assure accuracy in the results. For real analytes, like cancer cells, the test would involve calibration against known RIs and sensitivity benchmarks established through FEM simulations. Additional coating layers or protective casings would be a way to balance between robustness and performance. The result of these steps will validate the theoretical design of the sensor and will also provide the way for its practical application in cancerous cell detection. These observations demonstrate the promise of PCF‐based sensors in oncology, as their capability to differentiate between cell types based on RIs could lead to more precise and early cancer detection. This advancement holds the promise of revolutionizing cancer diagnostics and treatment, thereby improving patient outcomes. The production tolerance of the suggested THz PCF biosensor for cancer cell identification is enhanced by its highly optimized design that maximizes optical performance and ease of manufacturing. Periodicity of the structure, defined by a constant pitch (P), facilitates manufacturing by requiring uniform control over core and cladding dimensions. Materials like Zeonex, chosen for low loss of absorption and high optical transparency, also support manufacturing with good performance in the THz regime. State‐of‐the‐art lithography, stack‐and‐draw processes, and 3D microprinting technologies offer precise geometry for curved trapezoidal air holes and stacked cladding. Stringent quality control during fabrication ensures uniformity in dimensions, enabling consistent optical properties and reliable sensor operation.

**FIGURE 11:**
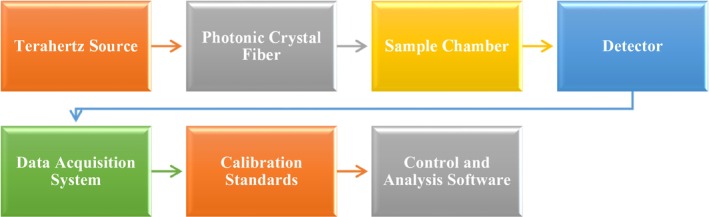
Provide a case study of the suggested PCF sensor's experimental procedure.

Figure 11 outlines a standard experimental setup involving essential components and stages to assess the effectiveness of the THz PCF sensor in detecting cancer cells professionally. The PCF sensor for cancer cell detection is described in this experimental protocol. THz radiation is first produced by a THz source and then directed via a PCF. After that, the waves go into the sample chamber that holds the biological sample, where they come into contact with it. The data collection system processes the information obtained from a detector that measures the variations in the THz waves. While control and analysis software analyze the data to examine and identify malignant cells based on the wave characteristics, calibration standards guarantee accuracy. THz sensing technologies and sophisticated data processing are used in this system to provide medical diagnoses.

Some future strategies that can be employed to enhance the performance of the sensor and extend its application areas include optimization of the circular core PCF design. For instance, fine‐tuning the geometrical parameters like air hole dimensions, spacing, and the pitch can be done for maximum light confinement and sensitivity. Advanced computational techniques such as machine learning and genetic algorithms could replace the trial‐and‐error approach in efficiently pinpointing the optimal configurations. Other areas of importance would be material innovation. Even though the material Zeonex is already very clear optically and has small absorption loss, further searches for advanced materials with good thermal stability, excellent biocompatibility, and mechanical strength can further ensure the sensor's reliability for wider application. Hybrid materials or coatings also enhance the selectivity to various cancer biomarkers; thus, enabling the detection of multiple cancer types using one sensor. For integration into portable diagnostic tools, it is necessary to miniaturize the sensor and integrate it with microfluidic systems for the manipulation of biological samples. Coupling with low‐power THz sources and detectors may enable on‐site non‐invasive testing. Cost‐effective manufacturing for widespread deployment can be made possible by the use of advanced manufacturing techniques such as 3D printing or scalable lithography that can replicate the intricate PCF structures with high accuracy at lower costs. This can be achieved by collaboration with industries and regulatory bodies to streamline the production pipeline while ensuring clinical standards.

## 5 Conclusion

Nowadays cancer has become a global plague, and the lives of patients are also being immensely threatened if not detected early. Therefore, localizing the precise location of cancer cells is essential for further progress. A comprehensive numerical study reveals that the suggested cancer cell detection sensor exhibits exceptionally high RS of approximately 99.60% for cervical cancer cells, 99.64% for adrenal gland cancer cells, 99.46% for skin cancer cells, 99.58% for blood cancer cells, 99.69% for breast cancer type I cells, and 99.71% for breast cancer type II cell detection with a minimal loss. In addition, the sensor provides very high NA values together with small spot sizes and EA that enhance strong light‐sample interactions. From the structure of the sensor, which is geometrical, it is quite easy to design and can easily be produced through any technique.

## Author Contributions

Conceptualization: A.H.M. Iftekharul Ferdous; Methodology: Kayab Khandakar, Md. Omar Faruk, A.H.M. Iftekharul Ferdous; Software: Kayab Khandakar, Md. Omar Faruk; Data curation: Kayab Khandakar, Md. Omar Faruk; Investigation: Kayab Khandakar, Md. Omar Faruk; Validation: A.H.M. Iftekharul Ferdous, Md. Naimur Rahman Naim; Formal analysis: A.H.M. Iftekharul Ferdous, Md. Naimur Rahman Naim; Supervision: A.H.M. Iftekharul Ferdous; Visualization: A.H.M. Iftekharul Ferdous, Md. Naimur Rahman Naim; Project administration: A.H.M. Iftekharul Ferdous; Resources: A.H.M. Iftekharul Ferdous, Md. Naimur Rahman Naim; Writing – original draft: Kayab Khandakar, Md. Omar Faruk; Writing – review and editing: A.H.M. Iftekharul Ferdous, Md. Naimur Rahman Naim.

## Ethics Statement

The authors have nothing to report.

## Consent

The authors have nothing to report.

## Conflicts of Interest

The authors declare no conflicts of interest.

## Data Availability

The data that support the findings of this study are available on request from the corresponding author. The data are not publicly available due to privacy or ethical restrictions.
